# Artificial Intelligence in Wound Care Education: Protocol for a Scoping Review

**DOI:** 10.3390/nursrep14010048

**Published:** 2024-03-14

**Authors:** Rúben Encarnação, Tânia Manuel, Hélder Palheira, João Neves-Amado, Paulo Alves

**Affiliations:** 1 Centre for Interdisciplinary Research in Health, Faculty of Health Sciences and Nursing, Universidade Católica Portuguesa, 4169-005 Porto, Portugal; tscarvalho@ucp.pt (T.M.); helder.palheira@hc-healthcare.com (H.P.); jamado@ucp.pt (J.N.-A.); pjalves@ucp.pt (P.A.); 2 Cardiology ICU, São João University Hospital Center, 4200-319 Porto, Portugal; 3 Prove.pt, Grupo Saúde Nuno Mendes, 4560-164 Penafiel, Portugal; 4 HC Healthcare & Innovation, 4445-176 Alfena, Portugal; 5 Cardiothoracic Center, São João University Hospital Center, 4200-319 Porto, Portugal

**Keywords:** artificial intelligence, wound, education, scoping review

## Abstract

As healthcare continues evolving in the age of digital technology, the integration of artificial intelligence has emerged as a powerful force, particularly in wound care. The education of healthcare professionals in wound care is crucial for ensuring they acquire the necessary knowledge and skills, optimizing patient outcomes. This paper outlines the protocol for a scoping review with the goal of mapping and analyzing the current scientific evidence regarding the potential impact of artificial intelligence in wound care education. The current protocol follows the JBI methodological framework. The search was conducted in December 2023 in the following databases: CINAHL Complete (via EBSCOhost), MEDLINE (via PubMed), Cochrane Library, Academic Search Complete, Scientific Electronic Library Online (Scielo), Scopus, and Web of Science. Electronics searches were conducted in the Scientific Open Access Scientific Repositories of Portugal (RCAAP) and ProQuest Dissertations and Theses, OpenAIRE, and Open Dissertations databases to access gray literature. Additionally, searches were performed on Google Scholar and specific journals such as the International Wound Journal, Skin Research and Technology, Journal of Wound Care, and Wound Repair and Regeneration. The initial database searches retrieved a total of 11,323 studies. After removing duplicates, a total of 6450 studies were submitted for screening. Currently, 15 studies are included in this review, and data charting and analysis are underway. The findings of this scoping review will likely provide insights into the application of artificial intelligence in wound care education.

## 1. Introduction

Wound care is an essential field of healthcare that involves the proper management and treatment of wounds to promote complete and timely healing and prevent complications that may lead to amputation, infection, and other potentially life-threatening outcomes. Traditionally, wound care has involved standard protocols, often relying on primary dressings and routine procedures. In recent years, innovative technologies, such as artificial intelligence (AI), have revolutionized wound care, providing more effective and efficient solutions for acute and chronic injuries.

The exact definition of AI remains a point of ongoing discussion. The term, created by John McCarthy in 1955, refers to the creation of computer systems able to perform tasks and solve problems that usually require human intelligence, such as image recognition, decision-making, and natural language processing [1,2,3,4]. It can be broadly defined as the incorporation of human intelligence into machines.

With the integration of AI-based technologies in daily life, applying such technologies will be indispensable for every organization. AI stands out as a highly promising technology within the healthcare sector. There is great optimism that these technologies have the potential to provide significant improvements in all healthcare domains [5,6,7,8,9,10]. Several AI functionalities are applied in the health sector, such as decision-making support, patient monitoring, early diagnosis, workflow improvement, information sharing, security, remote surgery, virtual patients, and virtual assistants [4,5,6,7,8,9,11].

Furthermore, AI has significantly transformed the training and education of professional healthcare personnel, offering numerous benefits. AI in education includes intelligent tutoring systems, chatbots, robots, and automated assessment tools integrated into digitized materials that improve education [4]. These advancements provide students with personalized, efficient, and immersive learning experiences, enhancing teachers’ understanding of students’ learning processes and enabling machine-supported queries at any time [4,12,13,14].

A significant feature of incorporating AI into medical education is its adaptive learning ability to analyze and offer instant feedback and assessment, allowing students to monitor their knowledge gaps, recognize weaknesses, and receive immediate guidance for improvement [4]. AI has the capability to tailor the learning experience based on each student’s individual needs, knowledge level, and preferred learning style, ensuring the delivery of relevant and efficient educational materials [12]. Educators can also gain advantages from analytics generated by AI, which offer assistance in recognizing trends and patterns in student performance, adapting teaching approaches as needed, and enhancing the overall learning experience. This can contribute to enhanced working efficiency and teaching competence.

Generative AI is defined as a form or subset of AI that uses machine learning and deep learning techniques to create new data. A crucial element of generative AI is the capability of understanding potential data distributions and producing new data that mirror the original set. This technology diverges from traditional AI tasks like classification or regression, as it is capable of autonomously generating new content, including images and text. Its applications extend to different areas; in image generation, Generative Adversarial Networks (GANs) are often used, while Recurrent Neural Networks (RNNs) and transformer networks are employed in natural language processing to produce novel textual content. This technology is also recognized for its potential to transform medical education [15,16].

The application of AI in education has drawn worldwide interest across numerous areas of healthcare professional education. Physicians and nurses have increasingly incorporated AI technology to enrich students’ learning experiences with more realistic, sophisticated, complex, and immersive simulations [17,18,19]. This enables students and healthcare professionals to refine their clinical skills within a secure and controlled learning environment [12]. The potential of generative AI in medical education can significantly enhance wound care training and practice. It offers innovative methods for personalized learning, cased-based learning and simulation-based training, continuous education, and research assistance [16].

Despite the various benefits and the potential of AI in medical education, some areas still require further investigation. These include clarifying the long-term implications of AI-driven learning methodologies for student performance, instructor–student interactions, and the ethical implications of AI [4,6,7,10,17,20,21].

Despite ongoing improvements in the wound care field, AI provides valuable contributions to early detection, risk factor analysis, risk stratification, prediction, diagnosis, intelligent treatment, outcome prediction, and prognosis evaluation [8,11,22,23,24,25,26]. From innovative dressings embedded with sensors to advanced imaging techniques, this technology is improving the healing process and supplying healthcare professionals with real-time data for decision-making support and predictive risk assessment related to wounds. This technology is transforming wound care from prevention to treatment, and wound care must adjust to this changing world to improve patient care [11,26].

Treating wounds is often a significant challenge for healthcare professionals, as the multitude of treatment criteria, care products, patient conditions, and responses complicate the healing process and results. To maximize patient outcomes in wound care, it is recommended that those involved possess the appropriate knowledge and skills [27,28]. Wound care education contributes significantly to patient well-being and healthcare efficiency.

While specific studies directly addressing the role of AI in wound care education are limited, emerging technologies are making significant progress in this area. For instance, the use of metaverse technology, including virtual reality (VR) simulations, provides immersive learning and training experiences that closely resemble real-life surgical scenarios, while also being more efficient in the use of resources [29,30]. Another advancement is the integration of Augmented Reality (AR) with machine learning (ML) algorithms, which facilitates real-time interventions and diagnostic information [29,30]. These developments in medical education highlight the growing integration of AI technologies, offering students realistic and immersive scenarios, especially in surgical fields, and this approach can be extended to wound care education [29,30]. The potential of combining these technologies with machine learning and AI indicates a promising future for more effective, personalized, and interactive learning experiences in healthcare education.

Using generative AI to create personalized quizzes or images of detailed and varied different wound types would enhance personalized and case-based learning, allowing students to diagnose and plan treatment for a wide range of wound conditions, and learning materials and feedback can be tailored specifically to wound care [31]. This approach would allow educators to address individual students’ strengths and weaknesses in wound care techniques, pathology, and patient management, for example.

The challenge lies in ensuring the academic integrity and validity of AI-generated research [31]. The impact on scholarly communication is profound, as it necessitates new methods for peer review and verification to maintain the credibility of the scientific process [31]. This emerging scenario presents both opportunities and challenges for academic communities.

In 1947, Alan Turing, a pioneering figure in computer science, delivered one of the earliest public lectures about computer intelligence, saying “What we want is a machine that can learn from experience” and the “possibility of letting the machine alter its own instructions provides the mechanism for this”. Integrating AI literacy into medical education curricula and rethinking assessment methods considering AI’s capabilities are essential for the rapid evolution of AI.

A preliminary search in the CINAHL Complete, MEDLINE (PubMed), Cochrane Database of Systematic Reviews, PROSPERO, and Open Science Framework (OSF) databases indicates that, at present, there are no published or ongoing scoping reviews or systematic reviews concerning the use of AI in wound care education. The literature refers to a considerable number of AI applications for wound care. However, the integration of AI into wound care education remains uncertain. Thus, the authors conducted a scoping review to map and analyze the existing scientific literature on the potential impact of AI in wound care education.

## 2. Methods

Given the limited knowledge about AI applications in wound care education and the emerging nature of this topic, a scoping review approach was determined to be the most appropriate method, given its purpose in mapping the evidence [32,33]. In addition, it emerged as the starting point for subsequent research [33,34]. Given their exploratory nature, scoping reviews are particularly useful when the goal is to map the existing literature on a broad topic, identify key concepts, and provide an overview of the available evidence [33,34,35].

Furthermore, unlike systematic reviews, scoping reviews provide greater flexibility in study selection, enabling a more expansive exploration of the literature [34,35]. This adaptability proves especially valuable in emerging fields with evolving evidence, where research questions may be less defined [34,35]. It aids researchers in understanding the current knowledge landscape, clarifying concepts, and shaping future research directions [35].

This exploratory approach makes them valuable for gaining a comprehensive understanding and mapping out this research area. This mapping can provide a detailed description of the available information on AI applications in wound care education, identifying possible gaps in knowledge, offering conclusions about the current state of research activity in this area, and making recommendations for future research.

This review protocol was registered in the Open Science Framework (OSF) platform (https://doi.org/10.17605/OSF.IO/MTGDX, accessed on 30 November 2023).

The scoping review follows the Joanna Briggs Institute’s (JBI) methodology for scoping reviews [33,36] and the Guidance for Conducting Systematic Scoping Reviews [33]. Results will be presented following the Preferred Reporting Items for Systematic and Meta-Analyses extension for Scoping Reviews (PRISMA-ScR) [32] guidelines.

### 2.1. Research Questions

It is suggested that scoping review questions should have a broad scope [37]. This involves delineating the concept, specifying the target population, and identifying health outcomes of interest to bring clarity to the scoping study’s focus and develop a robust search strategy [37]. The research questions were formulated through collaborative discussions with the research team and relevant stakeholders. A panel of experts, including advanced practice nurses, nurse researchers, and educators in the fields of digital health technologies and health education, were consulted to shape the research questions aligning with the objective of this review.

To achieve this study’s aims, the following research question was identified according to JBI recommendations in the PCC mnemonic guide: what evidence currently exists regarding the application of AI in wound care education?

Additionally, this review aims to answer the following sub-questions:

- How is AI integrated into wound care education?
- How is AI being used to educate healthcare students about wound assessment and management?
- How does AI contribute to the education of healthcare professionals in wound assessment and management, particularly in clinical and academic settings?
- What evidence currently exists regarding the use of machine learning and simulation technologies in AI-driven wound care education?
- What evidence currently exists regarding the application of AI in formal wound care educational programs and training?
- What are the benefits of AI application in wound care education for healthcare practitioners, students, and educators?
- What experiences and perceptions do healthcare practitioners, students, and educators have regarding AI in wound care education?
- What learning outcomes result from integrating AI technology in wound care education?
- What are the barriers to and facilitators of applying AI technology in wound care education?

### 2.2. Inclusion Criteria

To determine the main subjects under investigation and formulate the eligibility criteria, the PCC (Population, Concept, Context) framework was used:

- Participants: this study will encompass all literature that discussed participants as healthcare practitioners, students, and educators.
- Concept: this review will include literature that analyzes AI and its influence on wound care education.
- Context: education. To expand the scope of the review, the context will be broad and involve any educational settings without geographic restrictions.

The scope of the literature reviewed will include any quantitative, qualitative, and mixed-method studies. Additionally, gray literature (conference abstracts, theses, government reports, clinical practice guidelines, editorial and opinion papers) will be included as well. Provided they meet the eligibility criteria, this analysis may also include additional relevant manuscripts. Studies that do not explore AI in wound care education will be excluded.

Literature sources were limited to English, Portuguese, and Spanish, based on the authors’ language proficiency, without imposing geographical or cultural restrictions.

### 2.3. Search Strategy

We used the PCC method and field knowledge to identify relevant keywords concerning this topic. As recommended by JBI scoping review methodology [38], we performed preliminary research using keywords (artificial intelligence, wound, and education) on two databases relevant to the topic of interest: Medical Literature Analysis and Retrieval System Online (MEDLINE via PubMed) and the Cumulative Index to Nursing and Allied Health Literature (CINAHL via EBSCOhost). The titles, abstracts, and index terms of the identified studies were reviewed to extract the MeSH thesaurus and CINAHL Subject Headings used to describe the literature. Subsequently, in collaboration with a health sciences librarian, two reviewers developed the search strategy, which was peer-reviewed by the third expert reviewer based on the Peer Review of Electronic Search Strategies (PRESS) [32]. The MEDLINE (via Pubmed) search strategy can be found in Appendix A. The search strategy, including all identified keywords and index terms, was customized for each literature source. When available, subject headings such as MeSH and Emtree terms were used.

As experts in the development of scoping review protocols, team members reviewed the search terms, Boolean operators, and results to edit and enhance the search strategy.

Article reference lists were sourced for additional articles. This step aims to check for additional studies not previously identified. Establishing contact with the authors of the identified studies might be useful for potential clarifications or obtaining references.

The whole search was carried out in the following databases: CINAHL Complete (via EBSCOhost), MEDLINE (via PubMed), Cochrane Library, Academic Search Complete, Scientific Electronic Library Online (Scielo), Scopus, and Web of Science. Electronic searches were also conducted in the Scientific Open Access Scientific Repositories of Portugal (RCAAP) and ProQuest Dissertations and Theses, OpenAIRE, and Open Dissertations databases to access gray literature. Additionally, searches were performed on Google Scholar and specific journals such as the International Wound Journal, Skin Research and Technology, Journal of Wound Care, and Wound Repair and Regeneration. The selection of these databases was performed in collaboration with the health sciences librarian to guarantee comprehensive coverage of the key concepts (artificial intelligence, wounds, and education).

The searches were carried out on 1 December 2023, and all results were imported into Endnote vX20 (Clarivate Analytics, Philadelphia, PA, USA). Duplicated studies were subsequently removed.

### 2.4. Evidence Screening and Study Selection

In the first phase, article titles and abstracts were screened independently by two reviewers for eligibility criteria using the Rayyan QCR platform. In the second phase, potentially relevant records were obtained in full through institutional access or by emailing authors. The full text of potentially relevant evidence was screened according to the inclusion criteria by two independent reviewers.

To systematize the review and minimize research bias, two independent reviewers were involved in each selection phase. Any disagreements were addressed and resolved by reaching a consensus with a third reviewer until complete agreement was achieved. The review team conducted a pilot test of this process and held regular meetings to ensure consistency in the application of the eligibility criteria. During this process, 10% of the records were screened, and the results were compared and discussed with the team. Inclusion criteria were modified as needed.

The final scoping review will document the reasons for excluding studies that do not meet the inclusion criteria.

The methodological quality of the included studies will not be assessed. Scoping reviews, in contrast to systematic reviews, do not require the same degree of evidence since they do not synthesize results from sources through a formal appraisal process but instead aim to provide an overview of the literature [34].

The decision not to assess the methodological quality of the included studies is deliberate as it will allow us to conduct a comprehensive review that includes a broad range of relevant studies to address the research question and achieve the primary study objective. In scoping reviews, assessing critical appraisal or risk of bias is generally not recommended because the aim is to map the available evidence rather than provide a synthesized and clinically meaningful answer to a question [33,34]. Due to the diverse spectrum of study methods and interventions and the inclusion of both gray and published literature, conducting a critical appraisal was not feasible. Given the emerging nature of this topic, performing a methodological quality appraisal and subsequently excluding studies based on this evaluation may result in the rejection of relevant research. The decision not to conduct a quality assessment promotes transparency and rigor.

**Figure 1:**
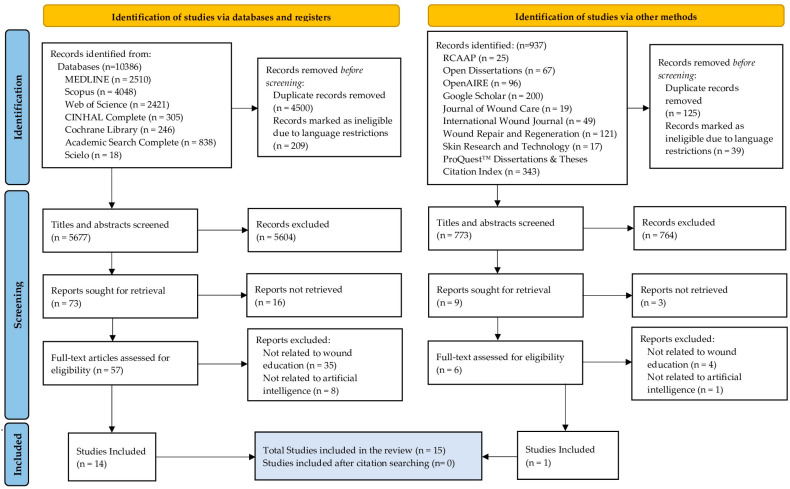
PRISMA (Preferred Reporting Items for Systematic Reviews and Meta-Analyses) 2020 flow diagram.

The research results will be fully described in the final review, and, at this stage, they are presented in a PRISMA-ScR flow diagram [39] (Figure 1).

### 2.5. Data Charting

Following the JBI methodology, data will be extracted from included records by two independent reviewers using a data extraction tool created by the review team based on the JBI instrument for extracting study details, characteristics, and results [33,34]. The authors will develop a pilot test of this form to ensure the appropriate capture of all relevant data. The initial three records extracted will be discussed and extracted data compared to assess any conflicts. Any disagreements between the reviewers will be resolved through discussion or with a third reviewer.

The extracted data contain specific details about the population (wounds), concept (artificial intelligence), context (education), and critical findings relevant to the review question and objective (Appendix B). This includes the title, author, year of publication, country of origin, research design, research purpose, participant details, wound specifics, AI characteristics, and education program details.

Participant details indicate the role (health student, health educator, or health practitioner). Regarding AI characteristics, the data include AI technology, tools, equipment, main functions, and other details (adaptive learning, interactive engagement, visual recognition, diagnostic assistance, feedback mechanism, continuous monitoring, and real-time updates). The data presented also encompass details of the education program, specifically the educational setting where it takes place (hospital, universities, and non-university settings), education level (undergraduate or post-graduate), and the outcomes measured.

It should be highlighted that adjustments to the data extraction tool could be carried out during the review stage. If there is any missing information in the included records, the respective authors will be contacted to request it.

### 2.6. Data Analysis

Data will be presented in a table and in narrative form to describe how the tabulated results relate to the review’s objective and question.

The same reviewers involved in the previous step will independently carry out this procedure. A third reviewer may be consulted to gain consensus on the differences found.

## 3. Results

A cohort of 15 studies has been earmarked for intricate data charting and analytical review. This selection follows a preliminary scrutiny of 6450 studies, distilled from an exhaustive retrieval of 11,323 potential studies.

The included papers were published between 2006 and 2023, encompassing a variety of study types, including conference papers, experimental studies, editorial papers, and other original articles. The majority of studies included in the review address various types of wounds, with some focusing specifically on certain types, such as pressure ulcers or diabetic foot ulcers.

The integration of AI in wound care education, as evidenced by these studies, marks a pivotal shift towards enhancing clinical decision support and e-learning platforms. This evolution is illuminated by these studies, which showcase AI’s capacity to transform healthcare education by offering tailored and engaging learning experiences. Moreover, these papers collectively underline the importance of evidence-based, personalized learning approaches facilitated by AI.

Several of these studies underscore the pivotal role of AI in fostering active learning and hands-on experiences. This is achieved through the development of e-learning scenarios that draw upon real-world experiences, thereby enriching the educational process with practical, applicable knowledge and insights.

The integration of AI paradigms, such as Bayesian Inference, Case-Based Reasoning, and Intelligent Agents, into e-learning platforms exemplifies a forward-thinking approach to medical education. This approach enables participants to analyze wound images based on color and texture, helping them to understand wound healing barriers such as non-viable tissue, infection, inflammation, and moisture imbalance.

Images entirely generated by AI to facilitate pattern recognition and clinical case discussions are also presented as a promising strategy to improve medical education. These AI-created visuals, not being real, carry the added benefit of preserving patient privacy while providing an innovative tool for educational purposes.

Other papers suggest employing smartphone-based AI applications for pressure injury assessment and utilizing case-based reasoning as educational tools. These methods enable learners to practice and enhance their knowledge and evidence levels, thereby maximizing their motivation and performance.

Large language models like ChatGPT are also presented as potential tools in providing personalized, accessible, and up-to-date educational content for healthcare professionals. This perspective underscores the flexibility and scalability of AI technologies in improving educational practices within the domain of wound care. It acknowledges, however, the necessity to address challenges pertaining to data privacy and the accuracy of the content provided.

In conclusion, the included studies indicate a promising future for AI in wound care education, pointing to the need for ongoing development, validation, and testing. Its relevance and pertinence to the research question are undeniable.

The comprehensive review will collate and present the outcomes within the main conceptual frameworks identified in this study. The authors will discuss and cross-validate the findings to ensure validity and credibility. They will also address the implications for future research, clinical practice, and policy while critically evaluating the significance of the findings concerning the study’s primary objective.

## 4. Discussion

As the World Health Organization (WHO) acknowledges, the use of AI in medical education presents a paradigm shift, offering unprecedented personalization and decision-making enhancements [40]. However, it also brings ethical dilemmas, such as ensuring patient privacy, fairness in AI-driven decisions, and legal accountability [40].

The WHO document on AI in health outlines several advantages and ethical pitfalls of AI in medical education. Some advantages are related to Enhanced Management and Diagnostics: AI aids in managing complex cases and streamlining routine diagnoses, enhancing the educational process with practical applications; Reduced Administrative Burden: AI reduces the workload on healthcare providers by handling clerical tasks, allowing more focus on education; Novel Insights from Data: AI provides new insights from health data, enriching educational content with advanced knowledge; and Support in Education and Research: AI tools support medical and nursing education and research, improving the understanding of medical conditions [40].

Despite this, we have as Potential Ethical Pitfalls the Quality of Education: AI inaccuracies could negatively impact medical education quality; the Additional Burden on Healthcare Workers: AI integration may require additional training for healthcare professionals not yet skilled in digital technologies; Bias and Privacy Concerns: AI in education raises issues of bias, privacy breaches, and accessibility, necessitating fairness and data protection; and Skills Degradation and Moral De-skilling: over-reliance on AI might erode clinicians’ skills and confidence in making independent decisions and moral judgments [40].

As emphasized by Drabiak et al., while AI and machine learning bring significant promise to education, particularly in personalizing learning experiences and enhancing educational efficiency, they also raise critical ethical concerns [41]. These concerns include issues related to data privacy, the responsibility of AI decision-making, trustworthiness of AI systems, and ensuring fairness in educational outcomes [41]. This perspective highlights the need for a balanced approach in AI implementation, ensuring that the technological advancements contribute positively to the educational landscape while conscientiously addressing ethical implications [41].

Incorporating AI into wound care education demands careful consideration of ethical aspects. The effectiveness of AI is also deeply influenced by the quality of the data it processes. For wound care education, it is essential to use high-quality, diverse, and representative data to avoid biases in AI algorithms. These biases can arise from both the data and human input, potentially distorting educational outcomes. Consequently, special attention to data selection and algorithm design is crucial for equitable and accurate educational experiences [41].

Furthermore, ethical aspects of AI, such as interpretability, accountability, and bias, are critical and need to be addressed carefully. Recent research has identified a need for better understandability of machine learning algorithms and predictions [42]. This includes clarifying AI’s role in decision-making, advocating for transparency, reducing algorithmic bias, and enhancing trust among stakeholders [42].

The integration of AI in wound care education should be ruled by robust ethical oversight. This involves establishing committees for the ethical review of AI applications, ensuring alignment with ethical principles, and respecting learner rights. Continuous assessment of the risks and benefits of AI in education is vital for maintaining integrity and trustworthiness.

Balancing the benefits of data sharing with the protection of individual privacy is a critical ethical challenge. Rigorous data security measures and transparent data usage tracking mechanisms are elemental. This approach helps maintain trust while leveraging AI’s educational advantages.

Transparency in AI operations and decision-making processes is also crucial in education. On the practical side, the implementation of AI faces technical and pedagogical hurdles. The cross-disciplinary nature of AI necessitates joint consideration of technical and legal aspects, especially in sensitive areas such as data cleaning in medical AI [19]. Instructors’ technological skills are instrumental but not sufficient for integrating AI in classrooms, further complicating the issue [19].

By addressing these ethical considerations, AI can be used in wound care education effectively, while also being ethically rigorous and responsible [43]. This approach enhances the educational experience while safeguarding the interests and rights of all involved stakeholders [43].

## 5. Limitations

We acknowledge potential limitations of the scoping review, particularly its restriction to studies published in English, Portuguese, and Spanish. However, no studies will be excluded based on country. Potentially relevant studies will be listed in a supplement of the final review.

The inclusion of diverse sources, with various study designs, may challenge the validity of the results. Variability in how AI and wound care education are defined and measured across studies may also affect construct validity.

While scoping reviews typically do not appraise the quality of included articles, and explicit reasons for not conducting such appraisals in this review are provided, this is acknowledged as another potential limitation of the scoping review. The lack of critical appraisal may limit the scoping review’s ability to provide concrete recommendations for practice or policy, as the quality of the included studies is not systematically evaluated. Despite these limitations, given that scoping reviews are considered a precursor to a systematic review [34], critical appraisal could be implemented if the conditions for conducting a systematic review are met.

Scoping reviews aim to offer a comprehensive overview rather than specific recommendations, limiting the ability to draw detailed conclusions and generalize findings. The applicability of results to various educational settings or populations might be constrained by the specific focus on AI in wound care education. Additionally, findings may not be directly transferable to different healthcare systems, educational institutions, or regions with distinct practices and resources.

We will consider the impact of these limitations and address any additional limitations that may arise when reporting our results.

## 6. Conclusions

AI plays a significant role in the future of healthcare education. Therefore, embracing AI and related technologies is not merely an option but a transformative trend that organizations must acknowledge and leverage for competitive advantage. While AI tools have been employed to assess wound care, efforts are needed to enhance their potential impact on wound care education. Integrating AI into health education, specifically wound care, represents a paradigm shift in how educational content is delivered and processed. This transformation goes beyond traditional methods to provide a more personalized, efficient, and interactive learning experience. AI-driven tools can adapt to individual learning styles and provide tailored teaching materials and simulations. This adaptability improves the learning process, making it more engaging and efficient for students and professionals alike. With AI’s ability to analyze and interpret complex medical data, students and physicians can gain insights into wound care from remote locations, breaking down geographic barriers in education. This is especially important in the current global situation, where distance learning and telemedicine are becoming increasingly important. We hope that integrating AI into wound care education will not only revolutionize the delivery of knowledge but also directly improve patient outcomes. By more effectively training healthcare professionals in wound care, AI can help better diagnose, treat, and manage wounds, ultimately improving the quality of patient care. This is consistent with the broader goals of health education, which focus on improving health outcomes and the standards of patient care.

As this scoping review will illustrate, harnessing the potential of AI in wound care education requires not only a thorough understanding of its capabilities but also a commitment to addressing the ethical and practical challenges associated with its implementation. A careful exploration of the ethical aspects surrounding artificial intelligence in wound care education is essential. As AI shapes the educational landscape, addressing issues like data privacy and bias is crucial for a responsible integration that ensures both educational excellence and ethical approval.

As far as we know, this is the first review to explore the influence of AI in the context of wound care education. The findings of this scoping review will likely provide insights into the application of AI in wound care education, identify research gaps in the literature, and promote further research initiatives. Hopefully, it will also be relevant for educators, students, policymakers, health and education organizations, and researchers in healthcare sciences and engineering.

The results will be disseminated through presentations at health education meetings and conferences and publication in a peer-reviewed health education journal.

## Data Availability

For data supporting reported results, please contact the authors of this review.

## Public Involvement Statement

There was no public involvement in any aspect of this research.

## Guidelines and Standards Statement

This scoping review follows the Joanna Briggs Institute’s (JBI) methodology for scoping reviews.

## Appendix A. Search Strategy Used for MEDLINE (via PubMed) Database

**Search**	**Query**	**Results**
#1	“wound*”[Title/Abstract] OR “ulcer*”[Title/Abstract] OR “bed sore*”[Title/Abstract] OR “bedsore*”[Title/Abstract] OR “pressure sore*”[Title/Abstract] OR “diabetic feet”[Title/Abstract] OR “diabetic foot”[Title/Abstract] OR “surgical dressing*”[Title/Abstract] OR “injury”[Title/Abstract] OR “injuries”[Title/Abstract] OR “pressure ulcer”[MeSH Terms] OR “wounds and injuries”[MeSH Terms] OR “surgical wound”[MeSH Terms] OR “wound healing”[MeSH Terms] OR “wound infection”[MeSH Terms] OR “varicose ulcer”[MeSH Terms] OR “ulcer”[MeSH Terms] OR “leg ulcer”[MeSH Terms] OR “skin ulcer”[MeSH Terms] OR “foot ulcer”[MeSH Terms] OR “diabetic foot”[MeSH Terms]	2,127,690
#2	“artificial Intelligence”[Title/Abstract] OR “AI”[Title/Abstract] OR “chatgtp”[Title/Abstract] OR “expert system*”[Title/Abstract] OR “computational intelligence”[Title/Abstract] OR “computer reasoning”[Title/Abstract] OR “computer vision”[Title/Abstract] OR “machine intelligence”[Title/Abstract] OR “machine learning”[Title/Abstract] OR “deep learning”[Title/Abstract] OR “natural language processing”[Title/Abstract] OR “neural network*”[Title/Abstract] OR “artificial Intelligence”[MeSH Terms] OR “Expert Systems”[MeSH Terms] OR “machine learning”[MeSH Terms] OR “deep learning”[MeSH Terms] OR “natural language processing”[MeSH Terms] OR “neural networks, computer”[MeSH Terms]	362,875
#3	“educat*”[Title/Abstract] OR “teach*”[Title/Abstract] OR “student*”[Title/Abstract] OR “training”[Title/Abstract] OR “instruction*”[Title/Abstract] OR “simulat*”[Title/Abstract] OR “interactive learning”[Title/Abstract] OR “gamification”[Title/Abstract] OR “game*”[Title/Abstract] OR “health education”[MeSH Terms] OR “teaching”[MeSH Terms] OR “learning”[MeSH Terms] OR “education”[MeSH Terms] OR “educational technology”[MeSH Terms] OR “simulation training”[MeSH Terms] OR “high fidelity simulation training”[MeSH Terms] OR “students”[MeSH Terms] OR “students, health occupations”[MeSH Terms] OR “students, medical”[MeSH Terms] OR “students, nursing”[MeSH Terms] OR “students, nursing”[MeSH Terms] OR “education, medical”[MeSH Terms] OR “education, medical, graduate”[MeSH Terms] OR “education, medical, undergraduate”[MeSH Terms] OR “education, graduate”[MeSH Terms] OR “education, medical, continuing”[MeSH Terms] OR “education, professional”[MeSH Terms] OR “health educators”[MeSH Terms] OR “educational personnel”[MeSH Terms] OR “faculty, medical”[MeSH Terms] OR “faculty, nursing”[MeSH Terms] OR “education, nursing”[MeSH Terms] OR “education, nursing, graduate”[MeSH Terms] OR “education, nursing, continuing”[MeSH Terms] OR “education, nursing, baccalaureate”[MeSH Terms] OR “education, continuing”[MeSH Terms]	3,242,478
#4	#1 AND #2 AND #3	2510
* includes truncated words in PUBMED.		

## Appendix B. Preliminary Data Charting Tool

Study characteristics	Extracted data
	Title
	Author(s)
	Publication year
	Country of origin
	Research purpose
	Research design
	Participants details (health practitioner, student, or educator)
Population	Type of wounds
Artificial intelligence characteristics	Technology (deep learning, expert system, machine learning)
	Tools, equipment and main functions
	AI details (adaptive learning, interactive engagement, visual recognition, diagnostic assistance, feedback mechanism, continuous monitoring, real-time updates, user-friendly interface, others)
	Evaluation metrics (to describe how the performance of AI technologies was measured)
Education program details	Domain (administration, assessment, learning, teaching)
	Educational level (undergraduate or post-graduate)
	Curricular structure and duration (to provide context regarding the scope and depth of the evaluated education programs)
	Outcomes of students
	Outcomes of teachers
Key findings	Relevant key findings
Clinical implications	To report how findings may influence real-life practice
Study limitations	To provide transparency and critical context
